# Thyrotoxic Periodic Paralysis: A Spine Consultation

**DOI:** 10.5435/JAAOSGlobal-D-18-00082

**Published:** 2019-11-04

**Authors:** Samuel J. Mease, Michael J. Faloon, Conor J. Dunn, Stuart Changoor, Nikhil Sahai, Awais K Hussain, Arash Emami

**Affiliations:** From the Department of Orthopaedic Surgery, St. Joseph's University Medical Center, Paterson, NJ (Dr. Mease, Dr. Faloon, Dr. Dunn, Dr. Changoor, Dr. Sahai, and Dr. Emami), and the Department of Orthopaedic Surgery, University of Illinois College of Medicine, Chicago, IL (Dr. Hussain).

## Abstract

As a consultant, the orthopaedic spine surgeon is often asked to evaluate patients with acute-onset extremity weakness. In some cases, patient's deficits can be attributed to nonspinal pathology; therefore, it is important to be aware of nonorthopaedic diagnoses when evaluating these patients. We report a case of thyrotoxic periodic paralysis that was initially confused by the consulting service with spinal pathology. A 32-year-old Hispanic man presented to our emergency department with rapid onset of lower extremity weakness. The consulting team ordered CT of the cervical and lumbar spine, as well as MRI of the lumbar spine which was aborted due to the patient's worsening tachycardia and chest pain. The spine service was subsequently consulted to evaluate the patient. Review of the metabolic panel revealed a low potassium, and additional testing led to the eventual diagnosis of thyrotoxic periodic paralysis. After correction of the patient's potassium, his weakness rapidly resolved, and no additional spinal workup was pursued. We describe this patient's presentation and outline the differential diagnosis for acute, nontraumatic extremity weakness, including both orthopaedic and other medical causes, that the spine surgeon should be aware of when evaluating patients with extremity weakness.

The acute presentation of extremity weakness is a potentially emergent situation, which may result in consultation of the spine service. Although there are many pathologies of spinal etiology (including compression due to hemorrhage, infection, neoplasm, or disk herniation) that may result in the sudden onset of weakness without inciting trauma, the examining orthopaedic surgeon or neurosurgeon should consider other nonspinal etiologies in their differential diagnosis. Thyrotoxic periodic paralysis (TPP) is one such condition that, while rare, can result in notable sequelae if unrecognized and untreated.

TPP is a condition characterized by thyrotoxicosis, hypokalemia, and paralysis, most often occurring in men of Asian or Hispanic descent.^1,2^ Although uncommon, it is increasingly reported in the United States because of the rising immigrant population. The paralysis associated with this condition most often affects the lower extremities. The hypokalemia results from an intracellular shift of potassium induced by thyroid hormone sensitization of Na+/K+ ATPase.^1^

It is important for clinicians to be aware of the pathology and presentation of TPP because it often manifests similarly to another condition, familial periodic paralysis. Misdiagnosis can result in delay of appropriate treatment. Although both conditions may present with paralysis and hypokalemia, subtle differences, such as no family history of paralysis, hyperthyroid symptoms, male sex, and presentation between second and fourth decades of life, can aid in the diagnosis of TPP. Prompt diagnosis allows for definitive management of TPP, which includes nonselective beta-blockers and correction of hyperthyroidism.^1,3^ The following case report demonstrates a case of TPP with lower extremity paralysis for which the orthopaedic spine service was consulted.

## Case Report

### Clinical Course

Our patient is a 32-year-old Hispanic man with no known medical history, who presented to the emergency department with a chief complaint of rapid onset of bilateral lower extremity weakness. He was initially evaluated by the emergency department physician and sent for a CT scan of his cervical and lumbar spine, as well as MRI of his lumbar spine. During the MRI study, he began to complain of severe substernal chest pain and became tachycardic and diaphoretic. Therefore, the study was terminated, with only sagittal T2 images having been obtained.

At this point, orthopaedic spine consultation was placed, and the patient was evaluated by the orthopaedic service. The patient was anxious appearing. He reported rapid progression of bilateral lower extremity weakness, since its onset the evening before presentation, from which he was no longer able to ambulate. He also reported heart palpitations, chest pain, and feelings of generalized anxiety. He denied any recent trauma, illness, or history of similar occurrence. He denied any paresthesias, fevers, chills, or constitutional symptoms.

### Examination, Laboratory Test Results, and Imaging

On examination, he had profound motor deficits in his bilateral lower extremities, with 0/5 muscle strength in the L2-S1 distribution. In the upper extremities, he also had evidence of profound weakness, with approximately 3/5 motor strength in the C5-T1 distributions. Sensation was intact globally. He was hyporeflexic in bilateral upper and lower extremities with no evidence of long tract signs including clonus or Hoffman's sign. Perianal sensation was intact, although the patient's rectal tone was decreased.

Laboratory evaluation revealed potassium of 1.6 milliequivalents per liter (mEq/L) (normal: 3.5 to 5.0 mEq/L), and phosphorous of 1.7 milligrams per deciliter (mg/dL) (normal: 2.5 to 4.5 mg/dL). There were no other notable electrolyte abnormalities. The patient's pulse was 139 beats per minute. ECG obtained after his aborted MRI demonstrated wide-QRS-complex tachycardia (QRS interval 164 milliseconds). Amiodarone was administered after which the patient's heart rate was well controlled and the QRS interval normalized. Subsequent thyroid function tests revealed hyperthyroidism and thyrotoxicosis (T4 = 18.4 micrograms per deciliter (μg/dL), T3 = 2.52 nanograms per milliliter (ng/mL), and thyroid stimulating hormone = 0.018 milliunits per liter (mU/mL)) (normal: T4 4.6 to 12 μg/dL; T3 0.8 to 2.0 ng/mL; TSH 0.4 to 4.0 mU/L, respectively).

The CT and MRI imaging studies of the patient's spine ordered before orthopaedic consultation demonstrated no evidence of compression or other notable abnormalities. Neurology and endocrinology consultation was recommended and obtained. Intravenous potassium was administered after which, on repeat evaluation by the orthopaedic team, the patient was found to have dramatic improvements of his previously appreciated motor deficits. Therefore, completion of MRI evaluation of the entire spine was deemed unnecessary, and care was deferred to medical subspecialties, with the orthopaedic team following closely.

### Management and Post–Emergency Department Course

The patient was diagnosed with TPP, and endocrinology consultation was obtained. As per recommendations of the endocrinology team, the patient was placed on methimazole (10 mg every 8 hours) and propranolol (40 mg every 6 hours). Methimazole was then changed to 20 mg twice daily. The patient was also started on dexamethasone (2 mg intravenously every 8 hours) because of its role in inhibiting peripheral conversion of thyroid hormone from T4 to the more active T3 form.^4^ On final evaluation before discharge, the patient had fully recovered all deficits.

## Discussion

This was a case of a 32-year-old Hispanic man who presented with acute-onset lower extremity weakness. The orthopaedic service was consulted for evaluation of the spine; however, imaging obtained before consultation demonstrated no evidence of acute spinal pathology. Laboratory studies demonstrated hypokalemia and elevated thyroid hormone, suggesting that the cause of the patient's weakness was not spinal in nature. The constellation of symptoms, as well as laboratory and imaging studies, eventually led to the diagnosis of TPP. The patient was treated with methimazole, propranolol, and dexamethasone, after which his presenting symptoms were completely resolved at the time of discharge.

TPP is a condition that occurs in 0.1% to 0.2% of the hyperthyroid population in North America.^5^ It occurs more often in men than in women and is more common in Asian and Hispanic individuals.^1,6^ The characteristic triad of findings associated with TPP includes hypokalemia, hyperthyroidism, and proximal muscle paralysis. Additional laboratory abnormalities that may be present in patients with TPP are hypomagnesemia and hypophosphatemia, which, along with hypokalemia, may also contribute to muscle weakness.^7-9^ The paralytic attacks associated with TPP occur mostly at night and are precipitated by alcohol, high carbohydrate diet, and heavy exercise.^10^ These characteristics can be considered when differentiating TPP from other causes of acute-onset extremity weakness.

The patient in this case was also found to have wide complex tachycardia at the time of presentation. ECG changes related to electrolyte abnormalities have been well described in the literature including ST segment depression with flattened T waves, sinus tachycardia, U waves, second-degree atrioventricular blocks, sinus arrest, ventricular fibrillation, and ventricular tachycardia.^11,12^ Thus, TPP represents a potentially life-threatening condition necessitating prompt diagnosis and treatment.

Unfortunately, the diagnosis of TPP is often delayed and can be confused with more familiar causes of paralysis.^2^ Such delays in diagnosis can be partially attributed to the subtlety of thyrotoxic symptoms and the unfamiliarity of the treating physician with this rare condition.^2^ Thus, it is not farfetched to suggest that the orthopaedic or neurosurgical spine service may be consulted by the emergency department for a patient with TPP, as was the case in our patient's initial management. Proper and prompt diagnosis through history, physical examination, and laboratory evaluation can prevent deterioration of the patient, unnecessary imaging, and allow the patient to receive appropriate treatment in a timely manner.

When evaluating a patient with acute extremity weakness, the spine surgeon's differential diagnoses should not only be limited to compressive spinal pathology but also include other etiologies, including inflammatory, neurodegenerative, and metabolic. Although spine surgeons may be confident in diagnosing some causes of weakness, mostly compressive in nature, they may not be comfortable with less commonly encountered conditions that may present without obvious compression on imaging. TPP is one such metabolic condition that presents with acute extremity weakness. Other conditions to be considered are myasthenia gravis, Guillain-Barré syndrome, and botulism.

Certain findings can help differentiate TPP from other conditions presenting with acute-onset weakness. In contrast to compressive causes of weakness, imaging studies in patients with TPP are normal. In TPP, proximal muscles are typically more severely affected than distal muscles. Mentation remains intact in patients with TPP, the respiratory muscles are spared, and the cranial nerves are not affected. Another important finding is that sensation is unaffected in TPP.^2,5,6^ Guillain-Barré syndrome is another cause of weakness that can be differentiated from TPP by its classic presentation of ascending paralysis and abnormal cerebrospinal fluid findings.^13^ Botulism is a toxin-mediated condition that presents as descending flaccid paralysis where the muscles of the head and neck are initially affected.^14^ In contrast to TPP, both Guillain-Barré syndrome and botulism can affect the respiratory system and lead to respiratory distress. Although neuromuscular disorders such as myasthenia gravis and Lambert-Eaton syndrome can present with extremity weakness, these are the result of an autoimmune reaction affecting the neuromuscular junction and do not typically present with electrolyte abnormalities.^15,16^ TPP is often misdiagnosed as familial hypokalemic periodic paralysis (FHPP) in Western countries because of similarities in precipitating factors and the clinical pattern of paralysis.^1^ However, TPP usually presents later in life with thyrotoxic symptoms and abnormal thyroid function tests. In contrast to FHPP, patients with TPP typically lack a family history of paralysis. In unclear cases, a urine calcium to phosphate ratio of >1.7 is a sensitive and specific test for diagnosing TPP and differentiating it from FHPP.^5^

In conclusion, although not a disorder of spinal etiology, it is imperative that spine surgeons are aware of TPP as it is a life-threatening condition associated with acute-onset extremity weakness for which they may be consulted. Key findings in the history, examination, and laboratory evaluation can aid in prompt diagnosis, obviating the need for expensive imaging. Prompt diagnosis reduces time to appropriate treatment, which includes nonselective beta-blockers, potassium repletion, and addressing the thyrotoxicosis. As the immigrant population in the United States continues to rise, so does the incidence of this uncommon disorder compelling spine specialists to be informed of TPP and to include it in their differential diagnosis.^17^
